# Medical interns’ reflections on their training in use of personal protective equipment

**DOI:** 10.1186/s12909-020-02238-7

**Published:** 2020-09-23

**Authors:** Ruth Barratt, Mary Wyer, Su-yin Hor, Gwendolyn L. Gilbert

**Affiliations:** 1 Centre for Infectious Diseases and Microbiology, Westmead Institute for Medical Research, 176 Hawkesbury Rd, Westmead, NSW 2145 Australia; 2 Marie Bashir Institute for Infectious Diseases and Biosecurity, University of Sydney, Sydney, NSW Australia; 3 Westmead Clinical School, Faculty of Medicine and Health, The University of Sydney, Sydney, NSW Australia; 4 Centre for Health Services Management, Faculty of Health, University of Technology Sydney, Sydney, Australia

**Keywords:** Personal protective equipment, curriculum., teaching., training., COVID-19., Infection control.

## Abstract

**Background:**

The current COVID-19 pandemic has demonstrated that personal protective equipment (PPE) is essential, to prevent the acquisition and transmission of infectious diseases, yet its use is often sub-optimal in the clinical setting. Training and education are important to ensure and sustain the safe and effective use of PPE by medical interns, but current methods are often inadequate in providing the relevant knowledge and skills. The purpose of this study was to explore medical graduates’ experiences of the use of PPE and identify opportunities for improvement in education and training programmes, to improve occupational and patient safety.

**Methods:**

This study was undertaken in 2018 in a large tertiary-care teaching hospital in Sydney, Australia, to explore medical interns’ self-reported experiences of PPE use, at the beginning of their internship. Reflexive groups were conducted immediately after theoretical and practical PPE training, during hospital orientation. Transcripts of recorded discussions were analysed, using a thematic approach that drew on the COM-B (capability, opportunity, motivation - behaviour) framework for behaviour.

**Results:**

80% of 90 eligible graduates participated. Many interns had not previously received formal training in the specific skills required for optimal PPE use and had developed potentially unsafe habits. Their experiences as medical students in clinical areas contrasted sharply with recommended practice taught at hospital orientation and impacted on their ability to cultivate correct PPE use.

**Conclusions:**

Undergraduate teaching should be consistent with best practice PPE use, and include practical training that embeds correct and safe practices.

## Background

One in ten patients acquire an infection associated with hospital admission [1], resulting in increased lengths of stay and morbidity [2, 3] and contributing to spread of antimicrobial resistance [4]. Healthcare workers can transmit pathogens and are themselves at risk of occupationally acquired infectious diseases including emerging/exotic viral infections such as COVID-19. The use of personal protective equipment (PPE) is a critical infection prevention and control (IPC) practice [5]. PPE protects the wearer and contributes to preventing further transmission, yet is often inappropriately used, especially by doctors [6, 7]. Many doctors feel that they have inadequate knowledge and practical skills, in IPC, generally, and PPE use, specifically [8], suggesting an unmet need for teaching undergraduates about its importance [9]. Mann and Wood found that around 50% of medical students thought their course should have had a greater emphasis on IPC [10], while John et al. reported that 92.5% of medical students made procedural errors when removing PPE during training [11]. In another study, Saudi Arabian medical students scored poorly on survey questions relating to PPE knowledge (average scores 3.8 ± 1.9 out of 9 points) [12]. In Hong Kong, the SARS outbreak exposed deficiencies in medical students’ PPE skills, which prompted changes in undergraduate IPC education [13].

Practical PPE skills are not always formally assessed at undergraduate level [11] and are often learnt via role modelling during clinical placements. The hidden curriculum therefore plays an important role in the clinical learning environment [14]. In relation to PPE use, interns may be influenced by the PPE practices of their role models – senior registrars and consultants – which can be suboptimal [15]. An understanding of the intern’s experiences and knowledge of the use of PPE at the beginning of their careers can help to inform undergraduate training and identify opportunities to enhance their skills in the clinical context.

Previous studies have used qualitative methods to investigate the influence of informal and hidden curricula in medical education [16, 17]; others have used self-reported surveys or observation [18, 19] - often focused on hand hygiene - to examine IPC knowledge and behaviours of medical students or junior doctors. In this qualitative study, we drew on discussions from reflexive focus groups to explore interns’ experience and behaviours related to the use of PPE. In this paper we report our analysis of these discussions.

## Methods

This study was undertaken in 2018 at a large tertiary-care teaching hospital in Sydney, Australia. This paper describes part of a larger study of medical interns’ previous experiences of personal protective equipment (PPE) training and use, including an evaluation of the likely efficacy and acceptability of video-reflexive methods for training.

Video-reflexive methods are derived from video-reflexive ethnography, a research methodology involving the videoing of everyday clinical work and showing the footage back to those videoed in guided reflexive groups to engender learning and change through collaborative and reflexive discussion [20]. We adapted video-reflexive methods for PPE training, by videoing medical interns during a practical PPE session (putting on and removing a full set of PPE), and showing them their footage during reflexive groups. The primary aim of this study was to compare the learning outcomes of interns who experienced the video-reflexive training, with those of a control group who experienced the same PPE training, but without the video component. Results of this study are in preparation.

All participants were engaged in reflexive group discussion following a PPE lecture and practice session. The only difference between the videoed and control groups was that the former were able to watch the footage of their own practical session during reflexive discussions. Consistent with the video-reflexive principle of foregrounding the complexity of everyday practice [20], both groups were asked open-ended questions about their experiences of PPE use prior to training, to understand their usual practices and contextualise what they had just learnt during training.

In this paper, we describe medical interns’ self-reported experiences of PPE use at the beginning of their internship, and explore the implications for future PPE training. The study was approved by the human research ethics committee of the relevant Local Health District.

### Setting and participants

Graduates of the 4 year postgraduate Doctor of Medicine (MD) programme at the Sydney Medical School (University of Sydney) are required to undertake an accredited internship to be eligible for registration as a medical practitioner. At our study site, new interns attend a 2-week hospital orientation which includes a 2 h session on IPC. The session involved a lecture and PPE practical session, which was the focus of this study.

In January 2018, all graduates commencing their internship at the study site were approached during hospital orientation as a convenience sample for the study. This type of non-probability sampling uses a population that is easily available [21]. Interns were informed about the research in advance and by email, by the Director of Prevocational Education and Training, and provided with a participant information sheet. At the start of the IPC session, the researchers (SH, MW, RB and GLG) described the study, distributed participant information sheets and consent forms and invited questions. After the lecture and immediately before the practical PPE session, researchers invited participants to opt in or out of the research, with reassurances that this would not affect their relationship with the hospital, none of their supervisors would know whether or not they had participated, and that those who did not participate would still receive the practical PPE session.

Seventy two of the 90 interns invited to participate accepted (80% response). The fact that 20% of interns felt able to decline suggests that they felt no sense of coercion, although those who participated may have felt some obligation to conform with the majority.

### Design

The four researchers who conducted the study are experienced in qualitative health research, and video-reflexive methods: they include two nurses with IPC expertise (RB, MW); an infectious diseases physician (GLG); and a social scientist (SH). The researchers were not employed by the hospital at the time of the study.

After a lecture from the hospital’s IPC department, including standard and transmission-based precautions and demonstration of the correct way to don and doff (put on and remove) PPE safely [22], consenting interns were allocated (using alternate selection) to either a control or video group. Both groups practiced donning and doffing gloves, gown, mask and goggles, after which they participated in reflexive group discussions, with each discussion led by one or two researchers. The only difference between groups was that those who were videoed were shown footage of themselves donning and doffing PPE during the discussions.

There were eight group discussions in total (four video and four control), each comprising 8–10 participants, and lasting 20–30 min per group. Discussions were audio-recorded and transcribed by an independent transcription service.

### Analysis

Two researchers (RB and MW) conducted a thematic analysis of the data in two stages, beginning with immersion through repeated readings of the transcripts, identifying themes (patterns of meaning) [23]. NVivo software (QSR International Pty Ltd. Version 12.6.0) was used to organise and code the data. The researchers (RB and MW) then matched these themes, independently, using the COM-B model [23] as a framework. The COM-B model is linked with the behaviour change wheel, allowing for analysis of determinants of current and desired PPE behaviour and identification of interventions that could affect behaviour change.

Participants’ statements about recent and prior PPE training and use were coded into the following behavioural conditions for appropriate PPE use in clinical practice: *capability* (knowledge and skills), *opportunity* (structural and environmental factors) and *motivation* (attitudes, habits, and decision-making). In this context, examples of *capability* include knowledge of when to use PPE, and skills and self-efficacy in donning and doffing PPE; examples of *opportunity* include access to PPE and social norms of the clinical unit; and examples of *motivation* include prompts for PPE use and the personal desire to protect oneself. Reliability of analysis was increased by comparing the coded data between the two researchers. Discrepancies were resolved through discussion. In this first stage of analysis, researchers searched for differences between video and control groups, but found similar themes between both groups. Therefore data were combined in the next stage of analysis.

In the second stage of data analysis, the COM-B themes were re-examined to identify overarching themes that impacted on participants’ PPE use in routine clinical practice. At this stage, a third, non-clinician researcher (SH), reviewed a sample of the transcripts against the codes and themes, to address any potential bias arising from the nurse researchers’ clinical and IPC perspectives.

## Results

Initial themes categorised using the COM-B framework can be seen in Table 1.

**Table 1:** Themes matched to COM-B categories

COM-B category	Themes
Capability	
Psychological capability	▪ Skills in donning and doffing PPE ▪ Understanding PPE principles ▪ Knowledge of PPE protocols/processes ▪ Undergraduate learning about PPE ▪ Prior clinical teaching about PPE
Motivation	
Automatic motivation	▪ Unconscious habit – e.g. unconsciously doffing PPE as learned in operating theatre
Reflective motivation	▪ Beliefs about benefits of PPE for self-protection
Opportunity	
Physical opportunity	▪ PPE availability/access ▪ Range of PPE sizes and different products ▪ Facility design
Social opportunity	▪ Peer behaviour ▪ Clinical team norms ▪ Role models ▪ Time constraints to don and doff PPE ▪ Interference with clinical assessment

At the second stage of analysis, two overarching themes were developed to describe factors that were likely to impact on interns’ safe and correct PPE use in clinical practice. These were: a) adequacy of prior knowledge and skills for practice; and b) using PPE in the real world. Within the COM-B framework, theme A corresponded with *capability/motivation,* and theme B with *opportunity.* Below, we describe these themes in more detail.

### Theme a: adequacy of prior knowledge and skills for practice

This theme describes participants' comments on their PPE knowledge and education *prior to* the training received that day. All participants indicated that they had received basic education on the use of PPE at medical school, and some had received additional PPE education or training during clinical placement (e.g. several who had had placements in the operating theatres had been taught how to don sterile gloves and gown). This prior learning had provided some PPE knowledge and skills which, after their brief IPC/PPE orientation training, participants recognised had not necessarily been adequate for safe PPE use. They reported that preparation for medical school practical exams included an emphasis on hand hygiene before patient contact, so they automatically performed hand hygiene prior to donning PPE. However, although they were taught that PPE is important, correct methods of donning and doffing were not always demonstrated or explained. As one participant described it: “We get told *what* to put on, but no one’s been, like, this is *how* you put it on...” (Participant 2 [P2], Focus Group 1 [FG1], *emphasis added.*)

Until then, many participants had been unaware of the risks of self-contamination during doffing or the rationale for the doffing steps that were taught. For example, most participants had been unaware that a critical step in doffing PPE safely was to perform hand hygiene *after* the removal of gloves (because of the potential to contaminate their hands [22]), as the following quotes suggest:

*“I’ve never washed my hands right after taking off the gloves.” (P2, FG4)*

*“We didn’t wash our hands after we took our gloves off.” (P1, FG6)*

Some participants had already developed incorrect and unsafe habits of PPE use. For example, some had previously tied their gowns at the front – where gown ties could become contaminated - and so risked contaminating their hands when doffing. One participant described their thoughts about the order of donning, during orientation PPE practice:

*“I was much more conscious of what I was doing, because when you’re on the ward, you’re just like, yeah, yeah, yeah... You think you’re doing it automatically, but now you [need to] think in steps.” (P1, FG2)*

Participants also recognised that their lack of understanding of correct donning and doffing of protective masks had led to unsafe mask use. For example, some had previously removed their masks upwards, over their face and hair, potentially contaminating themselves:

*“But yes, I didn’t realise that it was much safer to pull [the mask] … downwards.” (P4, FG1)*

There was also a notable knowledge gap in the use of eye protection, with many participants erroneously having believed that their own spectacles were an acceptable alternative to protective eyewear:

*“I would have my glasses on as well, so I would be like, I’m good.” (P6, FG3)*

For several participants, aspects of their PPE behaviour had been learnt in operating theatres, during surgical placements, which subsequently influenced their use of PPE in the wards. At orientation and during this study, PPE removal was taught according to current Australian guidelines [22], namely: remove gloves first, followed by hand hygiene, then remove eye protection, gown and mask (in that order). However, participants noted that, during the study, they had made a habit of removing their masks first, due to their experience in theatre:

*“I think it’s just habitual [to remove the mask first]… when you’re going into theatre, you put the mask on then the scrubs…” (P4, FG5)*

Also, many participants noted that they instinctively removed gown and gloves together in one motion as they had been taught to do in theatre:

*“I’d actually take it […] off like in theatres. Like I’d pull the gown off and then take it off with the gloves and pull it off as one unit.” (P1, FG7)*

Finally, participants demonstrated a mixed understanding of the items of PPE required for transmission-based precautions. Although some correctly identified the PPE required for contact, droplet and airborne modes of transmission, others expressed confusion about which type of mask to wear for various disease scenarios:

*“And like I said, there's a lot of misunderstanding about what each mask is used for. They just think this [N95] is the better mask. Use this mask, rather than, like, what is its actually for”.(P1, FG8)*

### Theme B: using PPE in the real world

Participants frequently referred to the differences between ‘real-life’ PPE use and how it was demonstrated at orientation. Factors contributing to these differences included the physical environment and resources, the behavioural norms of the clinical area where they worked and the expectations of their roles as junior doctors.

As taught in orientation training, the first step in donning PPE is to remove jewellery to allow for effective cleaning of hands and arms below the elbow. Participants described different approaches to this step across different clinical settings:

*“Obviously in surgery, you don’t have [jewellery] in surgical scrubs, but on the ward, everyone wears their watches and rings and stuff.” (P5, FG2)*

Conversley one participant described their experience working in a hospital where there was a strong emphasis on bare below the elbows:

*“Because at my hospital, they’re very diligent in making sure that below the elbows had to be, like, nothing, literally, … so that’s why a lot of us now are used to not wearing watches.” (P4, FG3)*

A commonly cited reason for non-compliance with removing jewellery on the ward was a requirement to use a watch for patient examination. There was also the practical barrier of finding a suitable, safe place for it when removed:

*“I actually physically take everything off, you know, and sit it on the sink. And hope that it’s still there when I go back.” (P1, FG5)*

*“[Removing jewellery is] difficult on a ward where you're going to lose your watch, yeah.” (P2, FG5)*

Another environmental barrier that impacted on PPE behaviour was the variability and availability of some PPE items in clinical areas. Different gowns and masks were available in different clinical settings, so could be unfamiliar. Participants particularly noted that goggles or protective eyewear were difficult to locate in wards and even when eye protection was available, it was not always suitable:

*“None of these goggles fit over my glasses.” (P1, FG4)*

One participant had addressed this problem by purchasing their own protective eyewear with prescriptive lenses. As medical students, participants had come to accept, as “normal”, that certain PPE items were never available on wards and, even if they were, they were rarely used anyway:

*“I don’t … usually find [protective eyewear] on the wards and most times when we do, everyone just wears the mask and gloves and gown, no-one wears goggles.” (P6, FG7)*

Participants identified that senior doctors are looked to as role models, but may not always model best practice, as described by the following participant:

*“When you’re a student who’s a bit less experienced, you’re just following what the rest of the team is doing and basing it off that… [but] they’re not often the best models to follow.” (P1, FG1)*

Frequently, as the most junior member of the team, the intern was often required to remain outside of the room during ward rounds, to write in the patient notes, which limited opportunities to practice donning and doffing skills. They also identified time pressure during ward rounds as a challenge to optimal PPE use, describing a lack of time for all team members to correctly don PPE:

*“I was in a rush doing ward rounds, so I just followed suit.” (P1, FG3)*

*“When you're on wards you're definitely pressured to do it faster because you want to go in. Whereas today [during the training] I was like, oh, I've got all the time in the world to go in and do it correctly.” (P3, FG6)*

## Discussion

This paper reports on reflexive group discussions conducted as part of a larger study, to investigate interns’ knowledge and experiences of the use of routine PPE at the start of their internship. We report on two overarching themes from our data which describe likely influences on interns’ safe and correct PPE use, namely a) interns’ previous knowledge and skills in PPE use (*capability and motivation*) and b) how real-life clinical contexts affect their PPE use *(opportunity)*. Participants’ comments suggest that different and multiple approaches to learning are needed. Classroom education needs to include more detailed explanation of the *logic* of PPE use, for standard and transmission-based precautions. In addition, greater emphasis on practical and reflective learning in situ is required during student placements, to ensure that interns can practice IPC safely once qualified.

The first theme illustrated discrepancies and gaps between correct PPE use and junior doctors’ behaviours, learnt during undergraduate placements. It showed a potential for personal risk to the clinician, if incorrect PPE was used or it was put on or taken off incorrectly. An important finding, not previously described, was that the habit of removing gown and gloves together, in one action, in the theatre environment, impacted on PPE behaviour during routine care. However, in consultation with the IPC team, we reasoned that the risk of self-contamination was no different between the method used in theatre and doffing gown and gloves separately (as per policy for routine care). Furthermore, this method is endorsed by the US Centers for Disease Control [24]. Another potential occupational risk, identified by participants, was that they rarely used goggles, safety glasses or visors and several mistakenly believed that their own glasses would protect their eyes from splash. This is a common misconception, as reported previously [25], consistent with previous reports of low rates (27–40%) of compliance with eye protection by medical staff [6] and reflected in the fact that they were often unavailable in the wards. However, eye protection over and above prescription glasses is still needed, when a risk of eye contamination with blood or respiratory secretions is anticipated, to prevent blood-borne [25] or respiratory viral infection [26].

In our study there was also a worrying knowledge deficit related to protective masks, which protect clinical staff from infections transmitted via respiratory droplets, such as influenza, meningococcal disease or via smaller aerosolised particles. For example, not all participants knew that they could be exposed to airborne infection such as pulmonary tuberculosis unless they wore a properly fitted particulate filter respirator (PFR). An understanding of appropriate facial protection has implications during emerging infectious disease outbreaks when transmission routes are unknown and both droplet (including eye protection) and airborne precautions (including PFR) may be required.

The current COVID-19 pandemic has led to a greater use of PFRs among healthcare workers than previously, and identified a need for more training in PPE skills [27]. The knowledge deficit identified in this study aligns with that of Peres et al. [28], in which surveys of advanced medical students and junior doctors found that 59% of respondents had inadequate knowledge about PPE. Potential self-contamination, due to errors in PPE doffing, has been demonstrated in a number of studies which has implications for pathogen transmission not only to clinicians, themselves, but also to the environment and patients [29, 30]. The current COVID-19 outbreak has highlighted the importance of correct PPE use to protect frontline workers [31].

Education and training designed for medical interns cannot assume that they arrive with an understanding of appropriate PPE principles and practice. Education and training programs should acknowledge and identify interns’ prior misconceptions and inappropriate practice, in order to address and correct them. These findings provide some indication of what those misconceptions and inappropriate practices might include. Ideally, medical school curricula should also include this training, since clinical placements begin early in graduate medical programs.

Several teaching methods have been used to bridge the gap between PPE theory and clinical practice, such as simulation and virtual reality [32, 33]. Video-reflexive ethnography has been more commonly used in research (and is necessarily based outside of the classroom), but has a track record in delivering practice change and improvements in IPC at the frontline [34, 35]. This is achieved through clinicians reflexively and collaboratively analysing video footage of their own practices. Our study tests an adaptation of video-reflexive methods, to improve the salience and sustainability of learning from classroom-based PPE training. Further adaptations of video-reflexive methods in classroom situations could be fruitful, particularly in combination with scenario-based simulation. However, as the second theme (discussed below) suggests, it may also be fruitful to incorporate more reflexive learning opportunities around PPE use during everyday clinical practice, with methods such as video-reflexive ethnography.

The second theme (b) captured the challenges faced by junior doctors, if they attempt to use PPE appropriately when working in clinical areas. Other team members who use PPE incorrectly, if at all, make it difficult for junior doctors to do so safely. This concurs with Cresswell and Monrouxe’s [36] finding that social pressures experienced by medical students and junior doctors are a barrier to optimal IPC practice. It illustrates the tension between what students learn in the classroom and are taught through the hidden curriculum in the clinical setting. Senior medical staff have a major influence on the use of PPE by junior medical staff, but there is a paucity of appropriate peer and/or senior role modelling [37].

However, frontline leadership can have a positive influence. Peponis et al [37] reported that real-time peer feedback significantly increased compliance with eye protection (from 25 to 44%, *p* = 0.0004) and protective masks (3 to 16%, *p* < 0.0001) by members of a trauma team. Others have shown leadership can improve hand hygiene compliance and central-line associated bacteraemia rates [38].

Our study also highlighted some of the practical challenges in implementing PPE policy in the clinical space. Participants described their being expected to scribe outside patients’ room, on ward rounds, as a constraint on learning PPE skills. Another challenge was time pressure during ward rounds, as previously reported [39, 40]. There were also potential conflicts related to other clinical skills. For example, participants reported that they could not observe a 'no jewellery' rule when they needed to use their watches for clinical assessments. Consistent with findings from other studies [6, 7], poor access to suitable PPE within clinical areas was an environmental barrier, contributing to lack of opportunity to develop appropriate skills. Unavailability of the correct sizes of gowns or masks increases the risk of self-contamination [25].

Application of the Behaviour Change Wheel [23] that accompanies the COM-B model would suggest that these barriers to ongoing skill maintenance and development would be more appropriately addressed by systemic organisational change, rather than relying on education or training. For instance, ensuring that appropriate PPE stocks are available, providing physical locations for staff to place items for temporary safekeeping, encouraging use of fob (rather than wrist-) watches for use in clinical assessment. Organisations also have a responsibility to ensure that senior medical staff fulfil their accepted professional responsibility to model appropriate clinical practice, including correct PPE use [41]. Nevertheless, we suggest that enabling reflexivity-in-practice, using methods such as video-reflexive ethnography, may not only identify these issues, but also allow clinicians to devise creative and sustainable strategies to address them, as they have previously in similar acute care settings [34, 42].

One limitation of this study is that the findings are confined to the experiences and practices of one cohort of interns within one local health district. Another is that participants’ comments may have been constrained by the group setting – for instance they may have refrained from describing experiences at odds with those expressed by fellow interns. They may also have refrained from sharing particular experiences or details specific to the medical profession with the non-medical (or non-clinical) researchers. However, the themes we identified were largely consistent with findings of previous studies, and are likely to be valid, if not necessarily complete. Exploring interns’ preparedness for optimal PPE use in other institutions and at other times, such as during the COVID-19 pandemic and afterwards, may identify different issues regarding their skills, knowledge and clinical experiences.

## Conclusions

This study has identified gaps in the knowledge and skills of medical interns related to optimal use of PPE and highlighted missed opportunities in the clinical area to reinforce safe practice. Medical students sometimes learn sub-optimal PPE use by poor role models and leaders which, unless corrected, is likely to become habituated as their careers progress. Junior doctors are more likely to work autonomously and be less exposed to peer support for PPE use than nursing staff; they generally see more patients, as they move between different wards and departments and so are more likely to be unwitting vectors of pathogen transmission within a facility [43].

Education and training in IPC, including appropriate use of PPE, for new doctors is important to minimise transmission of infection and prevent occupationally acquired infectious disease. Although many organisations provide generic IPC education at orientation for all clinical staff, including interns, the content and delivery of this training may not target gaps in prior knowledge and poor habits developed through experiences of PPE use as medical students, and does not routinely recognise the informal and hidden curriculum in medical education. Educators should incorporate these factors into training and education, by promotion of appropriate use of PPE that is situated in the everyday contexts of medically-orientated routine care such as ward rounds, and provides opportunities to reinforce donning and doffing skills. Our findings also indicate that organisations should incorporate interventions to address the contextual opportunity barriers, that go beyond education and training, in order to improve appropriate PPE use by junior doctors, and thereby reduce the risks of healthcare-associated infections for patients as well as staff.

## Data Availability

The datasets generated and/or analysed during the current study are not publicly available as individual privacy could be compromised but are available from the corresponding author on reasonable request.

## Abbreviations

PPE: Personal protective equipment
IPC: Infection prevention and control
